# Deletion Of XIAP reduces the severity of acute pancreatitis via regulation of cell death and nuclear factor-*κ*B activity

**DOI:** 10.1038/cddis.2017.70

**Published:** 2017-03-16

**Authors:** Yong Liu, Xiao-Dong Chen, Jiang Yu, Jun-Lin Chi, Fei-Wu Long, Hong-Wei Yang, Ke-Ling Chen, Zhao-Ying Lv, Bin Zhou, Zhi-Hai Peng, Xiao-Feng Sun, Yuan Li, Zong-Guang Zhou

**Affiliations:** 1 Institute of Digestive Surgery and State Key Laboratory of Biotherapy, West China Hospital, Sichuan University, Chengdu, China; 2 Department of Gastroenterological Surgery, West China Hospital, Sichuan University, Chengdu, China; 3 Department of General Surgery, Shanghai First People's Hospital, Shanghai Jiaotong University, Shanghai, China; 4 Department of Oncology, Department of Clinical and Experiment Medicine, Linköping University, Linköping, Sweden

## Abstract

Severe acute pancreatitis (SAP) still remains a clinical challenge, not only for its high mortality but the uncontrolled inflammatory progression from acute pancreatitis (AP) to SAP. Cell death, including apoptosis and necrosis are critical pathology of AP, since the severity of pancreatitis correlates directly with necrosis and inversely with apoptosis Therefore, regulation of cell death from necrosis to apoptosis may have practicably therapeutic value. X-linked inhibitor of apoptosis protein (XIAP) is the best characterized member of the inhibitor of apoptosis proteins (IAP) family, but its function in AP remains unclear. In the present study, we investigated the potential role of XIAP in regulation of cell death and inflammation during acute pancreatitis. The *in vivo* pancreatitis model was induced by the administration of cerulein with or without lipopolysaccharide (LPS) or by the administration of l-arginine in wild-type or XIAP-deficient mice, and *ex vivo* model was induced by the administration of cerulein+LPS in AR42J cell line following XIAP inhibition. The severity of acute pancreatitis was determined by serum amylase activity and histological grading. XIAP deletion on cell apoptosis, necrosis and inflammatory response were examined. Caspases activities, nuclear factor-*κ*B (NF-*κ*B) activation and receptor-interacting protein kinase1 (RIP1) degradation were assessed by western blot. Deletion of XIAP resulted in the reduction of amylase activity, decrease of NF-*κ*B activation and less release of TNF-*α* and IL-6, together with increased caspases activities and RIP1 degradation, leading to enhanced apoptosis and reduced necrosis in pancreatic acinar cells and ameliorated the severity of acute pancreatitis. Our results indicate that deletion of XIAP switches cell death away from necrosis to apoptosis and decreases the inflammatory response, effectively attenuating the severity of AP/SAP. The critical role of XIAP in cell death and inflammation suggests that inhibition of XIAP represents a potential therapeutic strategy for the treatment of acute pancreatitis.

Acute pancreatitis is an inflammatory disorder of the exocrine pancreas, which has a range of severity and causes considerable morbidity and mortality.^1^ Inflammation and parenchymal cell death are key pathological responses of pancreatitis.^2^ Although the underlying mechanisms have not been fully elucidated and there is no specific effective therapy, the disease is believed to originate in injured acinar cells, and uncontrolled inflammation also contributes to parenchymal necrosis.^2^ Pancreatic acinar cell death occurs principally via apoptosis or necrosis, with the apoptosis presumed to be predominantly protective,^3^ whereas necrosis elicits inflammation that can escalate systemically, causing distant organ damage and mortality.^4^

Indeed, the consequences of apoptosis and necrosis are distinct in acute pancreatitis, while the mechanisms underlying these two types of cell death are interrelated.^5, 6, 7, 8, 9^ Understanding the regulation of the two death pathways in acute pancreatitis is important because the severity of acute pancreatitis correlates with the extent of necrosis and inversely correlates with apoptosis.^2, 3, 5, 9^ That is, inhibition of apoptosis pathways leads to necrosis and increased severity of pancreatitis, whereas stimulation of apoptosis attenuates the severity of the disease. Therefore, revealing the key signaling molecules that determine the pattern of pancreatic acinar cell death (apoptosis versus necrosis) in pancreatitis will provide potential molecular targets for effective therapy in this disease.

The family of caspases is a major mediator of apoptosis in pancreatic acinar cells.^2^ There are two main apoptotic pathways. The death receptors and mitochondrial pathways are activated by caspase-8 and caspase-9, respectively. Activated caspase-8 and -9 subsequently cleave and activate the ‘effector' caspases, such as caspase-3 and caspase-7, which subsequently cleave intracellular substrates that cause apoptosis.^10, 11, 12^

X-linked inhibitor of apoptosis protein (XIAP) belongs to the inhibitor of apoptosis proteins (IAP) that represent a family of endogenous caspase inhibitors.^13^ Among others, the caspase inhibitory mechanism is best characterized for the XIAP. It contains three BIR domains and a RING domain.^14, 15^ Previous Biochemical and structural analyses of XIAP have determined that the linker preceding the BIR2 domain of XIAP directly blocks the active sites of caspase-3 and caspase-7,^16, 17^ while the BIR3 domain sterically hinders caspase-9 dimerization and its activation.^18^ In addition to caspases inhibition, a growing body of evidence exists to support a modulatory role for XIAP in NF-*κ*B activation. The BIR1 domain of XIAP directly interacts with TAB1 to induce NF-*κ*B activation. TAB1 is an upstream adaptor for the activation of the kinase TAK1, which in turn couples to the NF-*κ*B pathway. However, NF-*κ*B also has ability to transcriptionally activate the expression of XIAP.^19, 20^ NF-*κ*B activation is a key intracellular event in acute pancreatitis, and activation of NF-*κ*B in acinar cells has been reported to increase the severity of pancreatitis in mice.^21^ Thus, XIAP possibly can be a critical mediator during acute pancreatitis due to its regulative role on caspases activities and NF-*κ*B activity.

Necrosis was long regarded as an unregulated and uncontrollable process. Recent studies show that necrosis may occur in a regulated manner.^22^ So-called programmed necrosis (or necrosis-like programmed cell death) is mediated by death adaptor kinase such as receptor-interacting protein kinase1 (RIP1). RIP1 forms a death-signaling complex with the Fas-associated death domain and caspases in response to death domain receptor stimulation.^23, 24, 25, 26^ During apoptosis, RIP1 is cleaved/inactivated by caspase-3 and -8,^27^ it can also be regulated by XIAP.^28, 29^ The regulation of RIP1 has been suggested to be one of protective mechanisms against necrosis in cerulein-induced pancreatitis.^3, 30^

Despite these studies, the precise mechanisms underlying XIAP regulating cell death and inflammation in acute pancreatitis remains unclear. Our findings demonstrate that lack of XIAP promotes apoptosis and inhibits necrosis in acinar cells, decreases the pancreatic inflammatory response and ameliorates acute pancreatitis in mice through regulation of caspases, RIP1 and NF-*κ*B activation. This work improves our understanding of the complex role of XIAP in cell death and inflammatory response during acute experimental pancreatitis, which could provide the potential for the development of an innovative therapeutic approach for the management of acute pancreatitis.

## Results

### Effect of XIAP deletion on the severity of cerulein (with or without LPS)-induced pancreatitis

**Figure 1:**
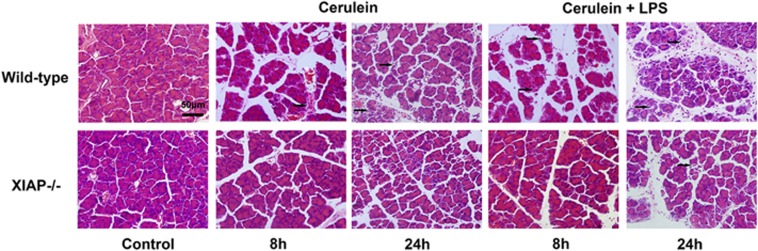
Effects of XIAP deletion on morphological changes in cerulein (with or without LPS)-induced pancreatitis. Results demonstrate a marked reduction in inflammation and acinar cell injury in pancreatic tissue from XIAP−/− mice induced by cerulein with or without LPS, as compared with wild-type mice. Controls were injected with saline alone in wild-type mice or in XIAP−/− mice. Bar indicates 50 *μ*m. Arrow shows the typical signs of pancreatic inflammatory pathology, including congestion and edema, cell death, leukocytes infiltration

Cerulein-treated mice displayed histological signs of acute pancreatitis characterized by interstitial edema, vacuolization and infiltration of neutrophil and mononuclear cells with little parenchyma necrosis and hemorrhage. The treatment of cerulein in combination with LPS caused more severe pathological changes in the pancreatic tissue, with an obvious edema, inflammation, vacuolization and a lot of local necrosis of acinar cells (Figure 1). In contrast, morphological changes seen in pancreatitis including acinar cell vacuolization, inflammation, edema and acinar cell necrosis were significantly less severe in XIAP−/− mice as compared with wild-type mice (Figure 1).

**Figure 2:**
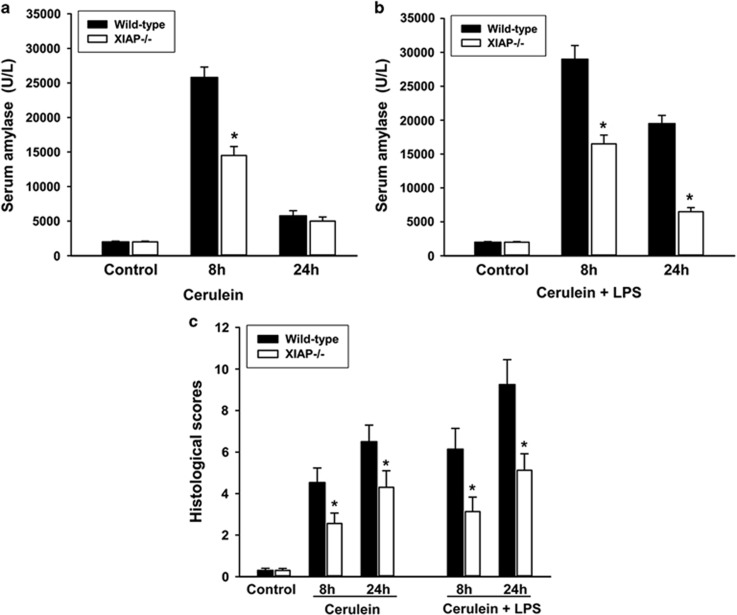
Effects of XIAP deletion on the severity of cerulein (with or without LPS)-induced pancreatitis. Results show that serum amylase (**a**, cerulein treated; **b**, cerulein+LPS treated), and histological scores (**c**) were all significantly decreased in the XIAP−/− mice, as compared with the wild-type mice. Results are expressed as means with the S.E.M. of at least three separate experiments with statistical significance at *P<0.05

The severity of pancreatic inflammation was further assessed by a semiquantitative scoring system, which was described in the ‘Materials and Methods' section. The results showed significantly decreased severity scores in the XIAP−/− mice as compared with wild-type mice, both in cerulein- and cerulein+LPS-treated groups (Figure 2c). Serum amylase was also used to quantify the severity of acute pancreatitis as described before.^31^ Treatment of wild-type mice by cerulein with or without LPS led to enhanced serum amylase levels compared with control mice. In contrast, serum amylase activities were decreased in the XIAP−/− mice as compared with the wild-type mice after cerulein as well as cerulein+LPS treatment (Figure 2a and b). In total, all parameters quantifying the severity of acute pancreatitis were reduced in the XIAP−/− mice as compared with the wild-type mice.

### Effects of XIAP deletion on inflammatory response in cerulein (with or without LPS)-induced pancreatitis

**Figure 3:**
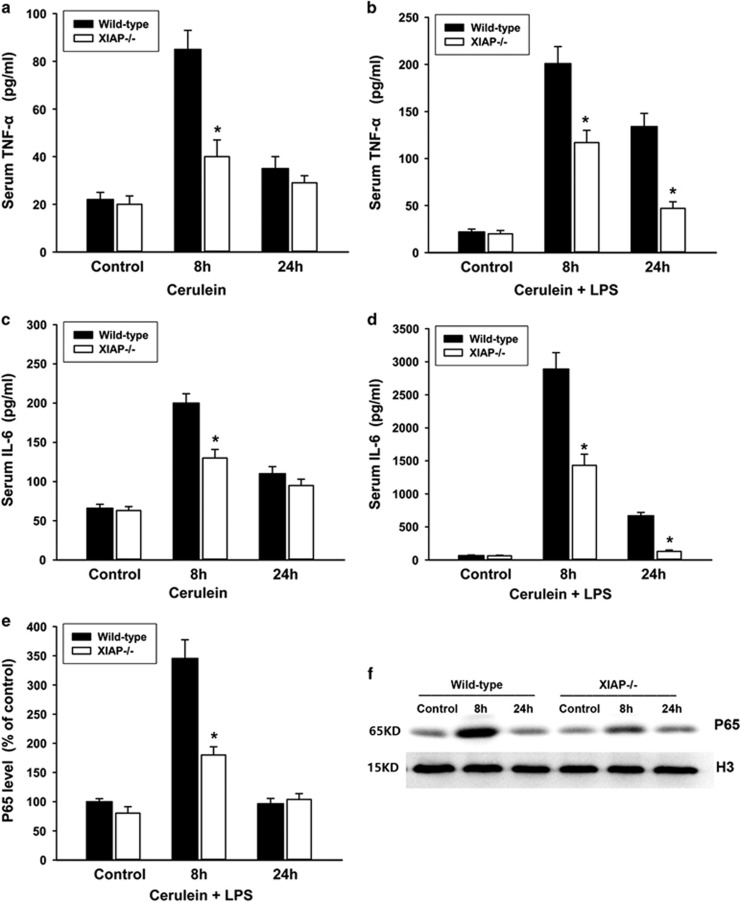
Effects of XIAP deletion on inflammatory cytokines and NF-*κ*B activation during cerulein (with or without LPS)-induced pancreatitis. The pro-inflammatory cytokines in the serum were determined by Luminex assay. Results demonstrate a slight reduction of TNF-*α* (**a**) and IL-6(**c**) levels in the serum from XIAP−/− mice induced by cerulein, as compared with the wild-type mice. Moreover, TNF-*α* (**b**) and IL-6 (**d**) were significantly decreased in the XIAP−/− mice induced by cerulein+LPS, as compared with the wild-type mice. The activation of NF-*κ*B was determined by detecting the levels of NF-*κ*B p65 subunit in the nucleus using western blotting (**e**). Quantification of p65 expression in pancreatic tissue from wild-type and XIAP−/− mice induced by cerulein+LPS was shown (**f**), data are expressed as percentage of the mean control value of wild type. Results are expressed as means with the S.E.M. of at least three separate experiments with statistical significance at *P<0.05

Increase of circulating pro-inflammatory cytokines is an important feature of acute pancreatitis, so in this study serum levels of TNF-*α* and IL-6 were determined. TNF-*α* and IL-6 serum levels revealed a pronounced increase after cerulein+LPS treatment compared with control animals. In contrast, this increase was significantly reduced in the XIAP−/− mice as compared with the wild-type mice (Figure 3b and d). Cerulein alone caused moderately elevated TNF-*α* and IL-6 levels in wild-type mice, and the moderate increases at early stage (8 h) were also reduced in the XIAP−/− mice (Figure 3a and c). In addition, we detected the activation of NF-*κ*B based on the levels of NF-*κ*B p65 subunit in the nucleus using western blot analysis (Figure 3f). The result revealed that cerulein+LPS induced significantly increased levels of NF-*κ*B p65 subunit at early stage (8 h) compared with control animals. In contrast, this increase was significantly reduced in the XIAP−/− mice as compared with the wild-type mice (Figure 3e).

### Effects of XIAP deletion on cell death in cerulein (with or without LPS)-induced pancreatitis

**Figure 4:**
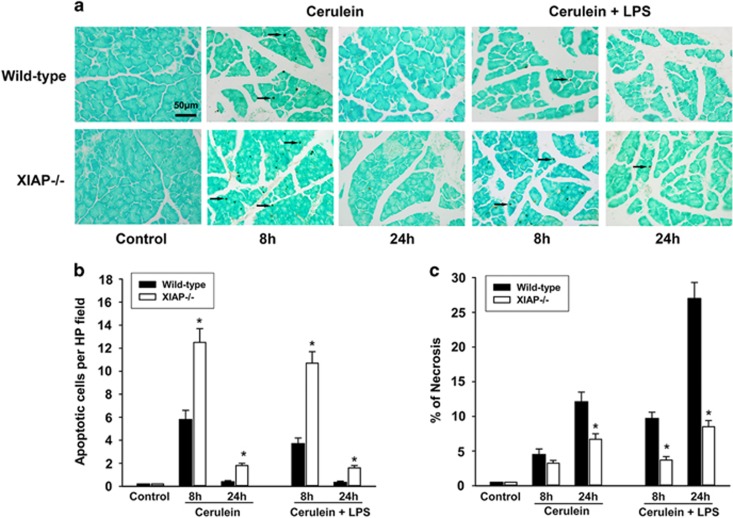
Effects of XIAP deletion on cell death during cerulein (with or without LPS)-induced pancreatitis. Apoptosis was determined by TUNEL assay (**a**), the results demonstrate a marked increase in apoptotic cells (**b**) in XIAP−/− mice, as compared with the wild-type mice treated by cerulein with or without LPS. Necrosis was measured on H&E-stained pancreatic tissue sections, the results show that necrosis (**c**) was significantly decreased in the XIAP−/− mice, as compared with the wild-type mice. Bar indicates 50 *μ*m. Arrow shows the positive staining of death cell. Results are expressed as means with the S.E.M. of at least three separate experiments with statistical significance at *P<0.05

To determine the role of XIAP deletion on cell death response in acute pancreatitis, we investigated the apoptosis during cerulein (with or without LPS)-induced pancreatitis (Figure 4a). The *in situ* terminal deoxynucleotidyl transferase-mediated dUTP-biotin nick-end labeling (TUNEL) assays showed that treatment of wild-type mice by cerulein with or without LPS both led to increased apoptosis at early stage (8 h) compared with control mice; nevertheless, no predominant apoptosis-positive cells were detected at late stage (24 h) after the first injection of cerulein. In contrast, this increase of apoptosis at early stage was significantly enhanced in the XIAP−/− mice as compared with wild-type mice, even that slightly increase of apoptosis-positive cells was detected at late stage in the XIAP−/− mice (Figure 4b).We also measured the effects of XIAP deletion on acinar cell necrosis during cerulein (with or without LPS)-induced pancreatitis. Morphological evidence of the extent of acinar cell necrosis was obtained by standard histological examination. The result showed that either cerulein or cerulein+LPS-induced acinar cell necrosis in XIAP−/− mice was significantly less than that in wild-type mice (Figure 4c), especially in the cerulein+LPS-induced group.

### Effects of XIAP deletion on activities of caspases in cerulein+LPS-induced pancreatitis

**Figure 5:**
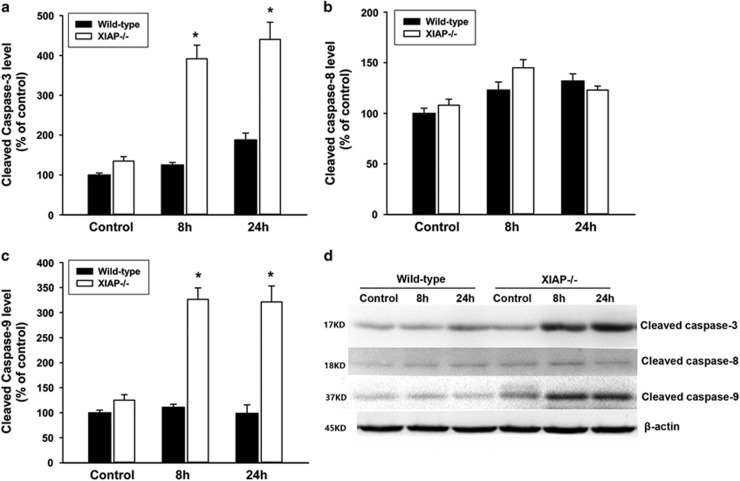
Effects of XIAP deletion on activities of caspases during cerulein+LPS-induced pancreatitis. The activities of caspases were measured by western blot analysis (**d**). Quantification of cleavage products of caspase-3 (**a**), caspase-8 (**b**) and caspase-9 (**c**) in pancreatic tissue from wild-type and XIAP−/− mice induced by cerulein+LPS were shown. Data are expressed as percentage of the mean control value of wild-type. Results are expressed as means with the S.E.M. of at least three separate experiments with statistical significance at *P<0.05 when compared between XIAP−/− mice and wild-type mice

We measured caspases activities in pancreatic tissue by western blot based on the cleaved antibody of caspase-3, caspase-8 and caspase-9. In wild-type mice, low levels of cleavage product of caspase-3, caspase-8 and caspase-9 were detected after cerulein+LPS treatment. In contrast, the levels of cleavage product of caspase-3 and caspase-9 expression were significantly increased in the XIAP−/− mice as compared with wild-type mice. However, the level of cleaved caspase-8 was no significant difference between XIAP−/− mice and wild-type mice (Figure 5). Thus, XIAP deletion promotes caspase-3 and caspase-9 activation, which mainly mediate the intrinsic apoptotic pathway, resulting in increased apoptosis during cerulein+LPS-induced pancreatitis.

### Effects of XIAP deletion on RIP1 degradation in cerulein+LPS-induced pancreatitis

**Figure 6:**
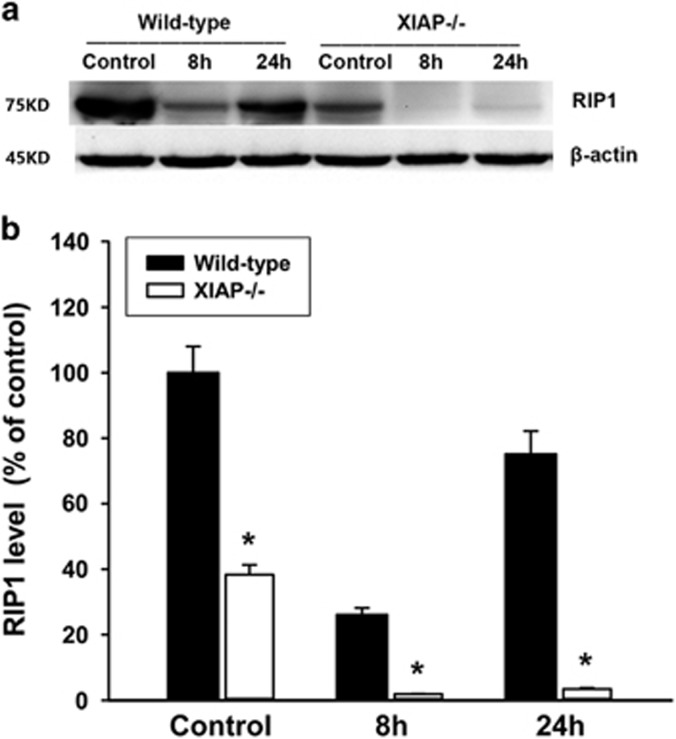
Effects of XIAP deletion on RIP1 expression during cerulein+LPS-induced pancreatitis. The protein expression of RIP1 was measured by western blot analysis (**a**). Quantification of RIP1 expression in pancreatic tissue from wild-type and XIAP−/− mice induced by cerulein+LPS was shown (**b**). Data are expressed as percentage of the mean control value of wild-type. Results are expressed as means with the S.E.M. of at least three separate experiments with statistical significance at *P<0.05 when comparison was made between XIAP−/− mice and wild-type mice

RIP1 has recently emerged as a key mediator of programmed necrosis in acute pancreatitis. In the present study, we examined whether XIAP regulates RIP1 degradation during acute pancreatitis. The result of western blot analysis showed that cerulein+LPS induced approximate 5-fold decrease in RIP1 expression at early stage (8 h) in wild-type mice. In contrast, the RIP1 expression level was significantly decreased as compared with control (only about 1/20) in XIAP−/− mice (Figure 6). These findings indicate that XIAP deletion promotes RIP1 degradation and inactivation, which resulted in less necrosis during the cerulein+LPS-induced pancreatitis.

### Alteration of XIAP expression regulated cell death pathways in pancreatic acinar cells

**Figure 7:**
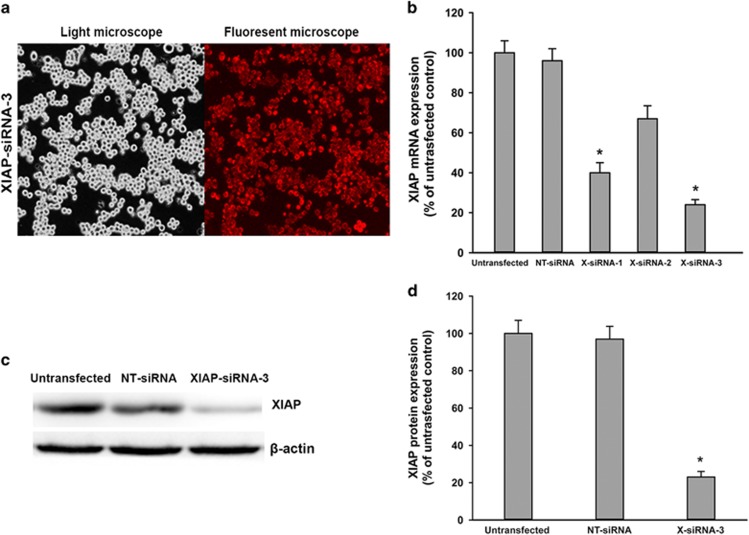
Effects of XIAP-siRNA on XIAP mRNA and protein expression in AR42J cells. The transfection efficiency of XIAP-siRNA-3 in AR42J cells were detected using light microscopy and fluorescent microscopy (**a**).The mRNA expression of XIAP was measured by real-time RT-PCR analysis (**b**). The protein expression of XIAP was measured by western blot (**c**), and quantification of XIAP protein expression was shown (**d**). Data are expressed as the percentage of the untreated control. Results are expressed as means with the S.E.M. of three separate experiments with statistical significance at *P<0.05 when comparison was made between XIAP-siRNA- and NT-siRNA-treated cells

**Figure 8:**
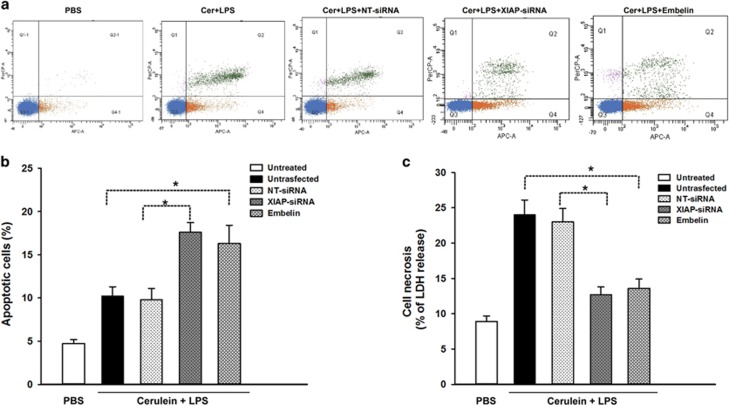
Effects of XIAP-siRNA or embelin on cell apoptosis and necrosis in cerulein+LPS-induced AR42J cells. The transfected cells or embelin-pretreated cells were incubated in F-12K medium for 24 h with cerulein+LPS. Cell apoptosis was measured by flow cytometry as shown (**a**), apoptotic cells are presented in the right lower quadrant of the figure (Q4), dead cells in the right upper quadrant (Q2), living cells in the left lower quadrant (Q3) and cell debris in the left upper quadrant (Q1). Results of analytical data show that the apoptotic cells were significantly increased in XIAP-siRNA-treated cells, as compared with NT-siRNA-treated cells. There was also a marked increase of apoptotic cells in embelin-pretreated cells as compared with untransfected cells. Cell necrosis was measured by the percentage of total cellular LDH released into the extracellular medium. Results demonstrated a marked reduction in LDH release (**b**) in XIAP-siRNA-treated cells or embelin-pretreated cells, as compared with NT-siRNA-treated cells or untransfected cells, respectively. Results are expressed as means with the S.E.M. of three separate experiments with statistical significance at *P<0.05

Although the *in vivo* e**x**periments shows that XIAP has a critical role in the regulation of cell death in pancreatitis, we further validate the function of XIAP by altering XIAP expression with molecular approaches in the cell culture model of pancreatic acinar AR42J cells. AR42J cells were transfected with pooled XIAP siRNAs (three duplexes), control cells received non-target (NT) siRNA. Twenty-four hours after transfection, highly efficient transfection of XIAP-siRNA in AR42J cells was documented with enhanced red fluorescent protein expression observed with a fluorescent microscope (Figure 7a). After 24 h incubation in cell culture medium, the XIAP mRNA and protein expressions were analyzed by western blot and real-time PCR (Figures 7b–d). The results confirmed that XIAP expression was best downregulated in XIAP-siRNA-3 transfected cells. We then measured cell necrosis and apoptosis after the transfected cells were incubated for 24 h with 100 nmol/l cerulein and 10 mg/l LPS. As shown in Figure 8, cell incubation with cerulein+LPS caused an increase in cellular LDH release (a measure of necrosis) in untransfected cells or NT-siRNA transfected cells (Figure 8c). Noticeably, knockdown of XIAP expression by XIAP-siRNA attenuated cerulein+LPS-induced cell necrosis. In contrast to its effect on necrosis, knockdown of XIAP caused dramatically increased cerulein+LPS-induced apoptosis determined by flow cytometry (Figure 8a and b). Furthermore, we use embelin (an inhibition of XIAP) at a concentration of 20 mmol/l to inhibit the function of XIAP. Inhibition of XIAP also affected the necrosis and apoptosis in cerulein+LPS-induced pancreatic acinar cells, with a similar trend as the effect of XIAP knockdown on cell death (Figure 8). Thus, we confirmed that downregulation or inhibition of XIAP promotes cell apoptosis while suppressing necrosis in pancreatic acinar cells.

### Effect of XIAP deletion on l-arginine-induced pancreatitis

**Figure 9:**
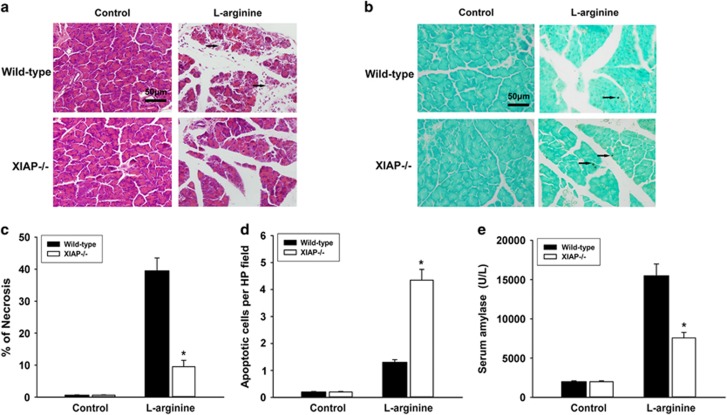
Effects of XIAP deletion in l-arginine-induced pancreatitis. Results demonstrate a marked reduced necrosis in XIAP−/− mice on hematoxylin and eosin staining (**a** and **c**), while an increase in acinar cell apoptosis in XIAP−/− mice on TUNEL assay (**b** and **d**), as compared with wild-type mice treated with l-arginine. Bar indicates 50 *μ*m. Arrow shows typical signs of pancreatic pathology (**a**) and the positive staining of death cell (**b**). Moreover, serum amylase (**e**) was significantly decreased in XIAP−/− mice, as compared with wild-type mice. Controls were injected with saline alone for wild-type mice and XIAP−/− mice. Results are expressed as means with the S.E.M. of at least three separate experiments with statistical significance at *P<0.05

To verify that the reduced effects of cerulein-induced acute pancreatitis seen in XIAP−/− mice were not acute pancreatitis model-specific, we examined the severity of pancreatitis induced in wild-type and XIAP−/− mice by l-arginine. Results show that there were significantly less serum amylase (Figure 9e), acinar cell necrosis (Figure 9c) and more acinar cell apoptosis (Figure 9b and d) in XIAP−/− mice treated with l-arginine as compared with wild-type mice. Morphological changes associated with acute pancreatitis were less severe in XIAP−/− mice as compared with wild-type mice (Figure 9a).

## Discussion

The development of acute pancreatitis is a complex process that is characterized by inflammation and parenchymal cell death.^32^ Cell death has different roles in different inflammatory diseases.^33, 34^ As for acute pancreatitis, the severity of inflammation correlates directly with necrosis and inversely with apoptosis, thus, shifting death responses from necrosis to apoptosis has a therapeutic value.^3^ Though evidences have shown that XIAP inhibits caspases^14, 35^ and induces NF-*κ*B activation,^20^ its role in the regulation of cell death and inflammatory response during acute pancreatitis remains unclear. It is necessary to study the specific effect of XIAP-related cell apoptosis in different diseases and to find out potential ways of treatment. In this study, we first induced acute pancreatitis in two different mouse models characterized by varying degrees of severity. Our results demonstrate that acute pancreatitis induced with cerulein alone shows a mild acute pancreatitis model as previously described.^36^ In contrast, acute pancreatitis induced by a combination of cerulein and LPS injection showed a relatively more severe model with deteriorated pancreatic inflammation, evident local acinar necrosis, as well as drastic systemic inflammatory responses, as previously reported. The effect of LPS on pancreatic tissue and inflammatory response have been evaluated, which showed intraperitoneal injection of LPS increases serum cytokine level but did not likely induce obvious pancreatic pathology, including cell death.^36, 37^ Importantly, all parameters quantifying the severity of acute pancreatitis were reduced in XIAP-deficiency mice as compared with wild-type mice, especially in the cerulein+LPS-induced model. Therefore, the model of cerulein+LPS-induced pancreatitis seemed more useful to study the role of XIAP in regulating cell death and inflammatory response during acute pancreatitis.

Nuclear factor *κ*B (NF-*κ*B) is an ubiquitous inducible transcription factor responsible for mediating the expression of hundreds of genes that have key roles in inflammation, immunity, cell death and proliferation, and is induced by a great variety of stresses.^38^ Recent studies have indicated that early pancreatic NF-*κ*B activation was found in all experimental models, mediated by degradation of both inhibitor kappa B (I*κ*B)-*α* and I*κ*B-*β*, and was associated with induction of cytokines and other inflammatory mediators.^39^ In most studies, pharmacological inhibition of NF-*κ*B activity ameliorated the inflammatory response, necrosis and other parameters of pancreatitis severity.^38^ Moreover, a recent study showed that increased acinar cell NF-*κ*B activity worsens acute pancreatitis through its effects on inflammation using novel genetic mouse models.^21^ These findings indicated that reducing the increased NF-*κ*B activity that occurs during acute pancreatitis likely would be beneficial for patients. In this study, cerulein+LPS induced significant NF-*κ*B activation at early stage, followed by enhanced pro-inflammatory cytokines such as TNF-*α* and IL-6 in wild-type mice. TNF-*α* was recently reported to have a dual role in regulating apoptosis during acute pancreatitis, a low concentration of TNF-*α* can induce apoptosis, whereas a high concentration causes acinar cell necrosis.^40, 41^ IL-6 has been shown to be elevated in experimental and clinical AP, with increased levels proportionate to increased severities of AP,^42, 43^ and there is evidence indicating that blockade of IL-6 can accelerate acinar cell apoptosis and attenuate the severity of a mouse model of acute pancreatitis induced by cerulein+LPS.^44^ Interestingly, our finding demonstrated that the activation of NF-*κ*B and levels of TNF-*α* and IL-6 are significantly reduced in XIAP-deficiency mice after cerulein+LPS treatment. In addition to caspases inhibition, XIAP is able to induce activation of NF-*κ*B.^20^ NF-*κ*B activation is known to increase the expression of the family of IAPs, including XIAP,^7^ and survivin^45^ and anti-apoptotic protein FLICE-inhibitory protein (c-FLIP)^46^ that inhibit the caspase system, the essential mediator of apoptotic death pathways. NF-*κ*B-dependent anti-apoptotic gene transcriptional activation has been demonstrated to be crucial in regulating cell death in pancreatitis.^2, 3, 47^ Thus, deletion of XIAP decreases the activation of NF-*κ*B, resulting in less release of inflammatory cytokines, decreased necrosis and increased apoptosis, all of which contribute to attenuate the severity of acute pancreatitis.

Apoptosis in pancreatic acinar cells is mediated mainly by activation of caspases. Of importance, it has been increasingly recognized that caspases not only mediate apoptosis but also protect from necrosis and decrease the severity of pancreatitis.^2, 3, 7^ XIAP is the most potent endogenous caspase inhibitor among the IAPs family, and it can inhibit mitochondria-driven caspases-3, -7 and -9.^16, 17, 19^ Thus, we suppose that block of XIAP can increase caspases activities and promote apoptosis in acute pancreatitis. In this study, we found that deletion of XIAP promotes cerulein+LPS-induced activation of caspase-3 and caspase-9, resulting in increased apoptosis, decreased necrosis and reduced severity of acute pancreatitis *in vivo*. On the other hand, either treated by XIAP inhibitor or downregulation of XIAP expression significantly increased apoptosis and reduced necrosis in cerulein+LPS-induced *in vitro* model of acute pancreatitis.

RIP1, a key regulator of programmed necrosis in many diseases including acute pancreatitis, has been known to be cleaved/inactivated by caspase-3 and -8,^23, 27^ and also regulated by XIAP.^28, 29^ Previous studies showed that enhanced protein levels of XIAP resulted in increased RIP1 production as well as decreased degradation, while genetic inhibition of XIAP expression markedly increased RIP1 degradation/inactivation in the pancreatic acinar cells.^48^ Our studies indicate that deletion of XIAP promotes the degradation/inactivation of RIP1 in cerulein+LPS-induced pancreatitis. It was also reported that RIP was cleaved by caspase-8 in TNF-induced apoptosis, and cleavage of RIP is an important process in apoptosis.^23^ On one hand, deletion of XIAP leads to more caspases activation, which cleaved the protein of RIP1, which resulted in inactivation of RIP1. On the other hand, downregulation of XIAP directly leads to increase of RIP1 degradation. Thus, increase of RIP1 degradation in pancreatic acinar cells resulted in less necrosis in both *in vitro* and *in vivo* models of acute pancreatitis.

To confirm that our findings were not unique to the models of cerulein-induced acute pancreatitis, we elicited a different model of acute pancreatitis induced by l-arginine. Hyperstimulation with cerulein or cerulein+LPS induces relatively mild or moderately severe acute pancreatitis, respectively. Although the l-arginine model is more severe and the succession of events associated with the acute pancreatitis induced by it occurs over a longer time span (peak injury occurred in 72 h),^49^ the result showed that necrosis is significantly greater in l-arginine-induced pancreatitis as compared with cerulein+LPS-induced pancreatitis. Deleting XIAP also significantly reduced necrosis and enhanced apoptosis in l-arginine-induced model of acute pancreatitis, though it is a little more effective in regulation of necrosis than apoptosis. These results establish that XIAP is a significant determinant of severity in pancreatitis, and its effect is not model-specific but common to all models tested.

In summary, our study demonstrates that XIAP is a key mediator of cell death in acute pancreatitis through its effects on caspases, RIP1 and NF-*κ*B. Lack of XIAP in acute pancreatitis decreases the activation of NF-*κ*B, increases caspases activities and RIP1 degradation, leading to less inflammatory response, increased apoptosis and decreased necrosis in acute pancreatitis, resulting in reduced severity of acute pancreatitis. Thus, lack of XIAP switches cell death away from necrosis to apoptosis and decreases the inflammatory response, attenuating the severity of acute pancreatitis. The critical role of XIAP in cell death and inflammatory response indicates that XIAP represents a potential therapeutic target in the management of acute pancreatitis.

## Materials and Methods

### Animals and reagents

All experiments were conducted with the approval of the Animal Research Committee at Sichuan University. The XIAP-deficient animals were obtained from Mutant Mouse Regional Resource Center (MMRRC) supported by National Institutes of Health (NIH, USA, strain name: B6; 129-Xiaptm1Thsn/Mmmh, stock number: 000021-MU). The XIAP-deficient mouse was backcrossed to the C57BL/6 J mice for at least six generations. Genotyping was performed by polymerase chain reaction (PCR). The animals were maintained on a 12 h light/12 h dark cycle at 22 °C, given water *ad libitum*, fed standard laboratory chow and allowed to acclimatize for a minimum of 1 week. The mice were randomly assigned to control or experimental groups, with four in the XIAP−/− group and four in the wild-type group at each time point, respectively. Cerulein, lipopolysaccharide (LPS), embelin (an inhibitor of XIAP) and medium F-12 K were purchased from Sigma Chemical (Sigma-Aldrich, St. Louis, MO, USA). AR42J cell line was from the American Type Culture Collection (ATCC; Manassas, VA, USA). Lipofectamine 2000, XIAP siRNAs and Non-Targeting siRNA Pool were from Invitrogen Life Technologies (Carlsbad, CA, USA). Antibodies against cleaved caspase-3, cleaved caspase-8, cleaved caspase-9, XIAP, RIP1, NF-*κ*B p65 subunit, histone H3.1 and *β*-actin were from Cell Signaling Technology (CST; Danvers, MA, USA). Other items were from standard suppliers or as indicated in text.

### Induction of experimental pancreatitis

For cerulein pancreatitis, the mice received seven intraperitoneal injections (IP) of 50 *μ*g/kg cerulein in saline, with a 1 h interval between injections.^50^ The second model of acute pancreatitis was induced by administration of cerulein in combination with LPS:^36^ the mice were injected intraperitoneally with cerulein in the same way as those in the cerulein acute pancreatitis model except that LPS was added (10 mg/kg) into the last cerulein injection. The mice were killed 8 and 24 h after the first injection of cerulein. The third model of AP was induced in mice by giving two intraperitoneal injections of l-arginine, each at concentrations of 4.0 g/kg body weight, with a 1 h interval between injections. The mice were killed 72 h after the second injection.^49^

### Cell culture and transfection of AR42J cells

AR42J cells were cultured in F-12 K medium supplemented with 20% fetal bovine serum at 37 °C with a humidified atmosphere containing 5% CO2. Transfection of AR42J cells with siRNAs was done using Lipofectamine 2000 (Invitrogen) according to the manufacturer's instructions. The plasmid constructs encoding XIAP siRNAs were applied in the transfection. The sequences of the three XIAP siRNAs (sense strands) are (siRNA-1: 5′-GCAAGAAGCUAUACGAAUG-3′ siRNA-2: 5′-CCGGAAUGUUAAUGUUCGA-3′ and siRNA-3: 5′-GCUUUAGGUGAAGGUGAUA-3′). The Non-Targeting siRNA Pool was used as controls. Since successfully transfected, the AR42J cells expressed red fluorescent protein, and the transfection efficiencies of XIAP siRNAs were observed using an Olympus IX81 fluorescent microscope. Forty-eight hours after transfection, the cells were used for experiments. The effectiveness of siRNAs in inhibiting XIAP expression was evaluated by real-time RT-PCR and western blot.

### Biochemical assay

Serum amylase was determined by means of a commercially available kit (R&D Systems, Minneapolis, MN, USA), and expressed as units per liter (U/l).The cytokines TNF-*α* and IL-6 in serum were measured using Luminex assay kit according to the manufacturer's instructions (R&D Systems). Assays were performed in duplicate using the Luminex 100 System (Austin, TX, USA). Individual cytokines were identified and classified by red laser, and levels were quantified using green laser. Digital images of the bead array were captured following laser excitation and were processed on a computer work station. Standard curves and reports of unknown samples were prepared using Master QT software (MiraiBio, Alameda, CA, USA).

### Histological examination

For light microscopy, fresh specimens of murine pancreas were fixed in 4% paraformaldehyde in phosphate-buffered saline (PBS, pH 7.4). The tissues were embedded in paraffin, and 5 mm sections were processed for hematoxylin and eosin (H&E) staining by standard procedures. Multiple randomly chosen microscopic fields from at least three mice in each group were examined and scored by two pathologists in a blind manner based on the presence of vacuolization, interstitial edema, interstitial inflammation, the number of acinar cell necroses, as previously described.^37, 51^ The scoring assessment was performed on a scale of 0–3 (0 being normal and 3 being severe) on each parameter mentioned above, and the sum of the scores were used to evaluate the severity of acute pancreatitis.

### Quantification of apoptosis

Apoptosis was quantified on the pancreatic tissue sections by the TUNEL assay. Briefly, the tissues were fixed in 4% buffered formaldehyde, embedded in paraffin and 5 mm-thick sections were adhered to glass slides. The sections were stained using TUNEL according to the manufacturer's protocol (Merck, Kenilworth, NJ, USA). TUNEL-positive cells (containing labeled DNA fragments) showed dark brown staining of the nucleus, suggesting the internucleosomal cleavage of DNA. The sections were counterstained with 0.3 percent methyl green. The numbers of positive apoptotic cells were counted in 10 high-power fields ( × 400 magnification), as described previously.^44^ Apoptosis of transfected AR42J cell culture was also quantified by flow cytometry using Cell Death Detection kit (BD Biosciences, San Jose, CA, USA) according to the manufacturer's instructions as described previously.^48^ Briefly, the cells were washed twice with PBS at 4 °C, re-suspended in 250 *μ*l 1 × binding buffer, and the cell concentration was adjusted to 1 × 10^5^/ml. APC Annexin V (5 *μ*l) and 7-AAD (5 *μ*l) were added and gently vortexed. After incubation in the dark for 15 min at 25°C, the cells were analyzed using a FACScan flow cytometer (BD Biosciences).

### Quantification of necrosis

Necrosis in mouse models of pancreatitis was quantified on pancreatic tissue sections stained with H&E. Cells with swollen cytoplasm, loss of plasma membrane integrity and leakage of organelles into the interstitium were considered to be necrotic, as previously described.^3, 47^ Necrosis in AR42J cells was determined by the release of lactate dehydrogenase (LDH) into the incubation medium, as previously described,^52, 53^ LDH activity was measured using LDH-Cytotoxicity Assay Kit (Sigma-Aldrich) according to the manufacturer's protocol.

### Real-time reverse transcriptase PCR

Total RNA was extracted from acinar cells using TRIzol (Invitrogen), followed by reverse transcription with a DNA reverse transcription system (Invitrogen). PCR was subsequently performed as described previously.^40^ The specific primers of XIAP were as follows: 5′-TGTGAGTGCTCAGAAAGATAAT-3′ (F) and 5′-TGCTTCTGCACACTGTTTACA-3′(R). *β*-actin was included in each reaction as an internal standard, and relative quantitative gene expression was calculated using the 2^−△△Ct^ method.^54^ Each sample was analyzed in triplicate.

### Western blot

For western blot analyses, portions of frozen pancreas tissue was rapidly homogenized in liquid nitrogen or acinar cells were isolated. Total protein and nuclear protein were extracted separately using the total Protein Extraction Kit (Sigma) and Nuclear Protein Extraction Kit (Viagene Biotech, Ningbo, China) according to the manufacturer's instructions. Nuclear protein extracts were used to detect the NF-*κ*B p65 subunit and histone H3.1. The concentrations of protein were determined using the BCA method (Pierce, Rockford, IL, USA). Each 20 *μ*g aliquot of total protein or nuclear protein was loaded in a 12% sodium dodecyl sulfate-polyacrylamide gel electrophoresis gel, and then transferred onto polyvinylidene difluoride membranes (Millipore, Billerica, MA, USA). After complete protein transfer, the membranes were blocked with 5% milk powder solution for 1 h at room temperature and incubated at 4 °C overnight with rabbit monoclonal anti-caspase-3, anti-caspase-8, anti-caspase-9, anti-XIAP, anti-RIP1 antibody and anti-NF-*κ*B p65 subunit diluted at a 1:1000 dilution in 5% milk powder solution. For internal reference, a rabbit monoclonal anti-*β*-actin antibody (1:1000 dilution) or anti- histone H3.1 antibody (1:1000 dilution) was used. After washing the membranes, goat polyclonal anti-rabbit immunoglobulin G secondary antibody (Cell Signaling Technology) conjugated to horseradish peroxidase was applied in a 1:5000 dilution and incubated for 1 h at room temperature. Finally, antibody binding was visualized using the enhanced chemiluminescence system (Pierce).

### Statistical analysis

The results are expressed as means±S.E.M. The data were analyzed using one-way analysis of variance with the Tukey–Kramer *post hoc* test. A *P*-value <0.05 was considered significant.
